# Thrombopoietin-receptor agonists for adult patients with immune thrombocytopenia: a narrative review and an approach for managing patients fasting intermittently

**DOI:** 10.3389/fcvm.2023.1260487

**Published:** 2023-12-14

**Authors:** Mohamed A. Yassin, Mona Al-Rasheed, Murtadha Al-Khaboori, Mahmoud Marashi, Hani Osman, Yasser Wali, Salam Al Kindi, Faisal Alsayegh, Drew Provan

**Affiliations:** ^1^ National Center for Cancer Care and Research, Hematology Section, Hamad Medical Corporation, Doha, Qatar; ^2^ Hematology Unit, Department of Medicine, Al-Adan Hospital, Hadiya, Kuwait; ^3^ Department of Hematology, Sultan Qaboos University, Muscat, Oman; ^4^ Dubai Academic Health Corporation, Dubai, United Arab Emirates; ^5^ Hematology-Oncology Department, Tawam Hospital, Abu Dhabi, United Arab Emirates; ^6^ Department of Child Health, Sultan Qaboos University, Muscat, Oman; ^7^ Faculty of Medicine, Department of Medicine, Health Sciences Center, Kuwait University, Kuwait City, Kuwait; ^8^ Academic Haematology Unit, Blizard Institute, Barts and The London School of Medicine and Dentistry, London, United Kingdom

**Keywords:** thrombocytopenia, thrombopoietin-receptor agonist, avatrombopag, eltrombopag, romiplostim

## Abstract

**Introduction:**

Thrombopoietin-receptor agonist (TPO-RAs) currently represent the state of art for treating immune thrombocytopenia. Their different molecular structures contribute to the difference in their pharmacodynamics and pharmacokinetics. This narrative review aims to provide an overview of the current TPO-RAs approved for primary immune thrombocytopenia (romiplostim, eltrombopag, avatrombopag) and the effect of intermittent fasting in adult patients receiving TPO-RAs.

**Areas covered:**

Literature was searched with no limits on date or language, using various combinations of keywords. Data on the pharmacokinetics, pharmacodynamics, efficacy, and safety of TPO-RAs and the effect of intermittent fasting were summarized.

**Expert opinion:**

Switching between TPO-RAs is a useful strategy to tackle some associated limitations. Romiplostim and avatrombopag have an advantage over eltrombopag as they do not require any dietary restrictions. In cases where romiplostim and avatrombopag are unavailable, patients should be educated on the appropriate administration, possible interactions, and dietary restrictions before initiating eltrombopag.

## 1. Introduction

Immune thrombocytopenia (ITP) is an autoimmune disease characterized by transient or persistent thrombocytopenia (platelet count <100 × 10^9^/L) and increased bleeding tendency (1). The incidence of ITP among adults was estimated to be 3.3 per 100,000 adults per year (2). The clinical picture ranges from asymptomatic mild thrombocytopenia to severe life-threatening bleeding, including intracerebral hemorrhage (3). In addition, patients may present with petechiae (on the skin or mucus membranes), ecchymoses, and oral mucosal blood blisters (3). Moreover, patients with ITP often report fatigue and impaired health-related quality of life (HRQoL) (4).

The mechanisms of ITP are complicated and not well-understood (5). One proposed pathogenic mechanism is that the antibody-coated platelets are prematurely destroyed in the spleen, liver, or both (6). Another pathogenic mechanism suggests that autoantibodies can induce complement-mediated or desialylation-induced destruction of platelets (7, 8) and inhibit megakaryocyte function (9).

The ITP World Impact Survey (iWISh), an exploratory survey focusing on the impact of ITP on HRQoL from patient and physician perspectives, demonstrated that ITP has a substantial impact on HRQoL (10). Patients reported that ITP reduced their energy levels and productivity. In the same context, physicians defined three main treatment goals for ITP patients: reduced spontaneous bleeding, improved quality of life, and healthy blood counts (11). The goals of treatment of ITP recommended by the International Consensus Report on the investigation and management of ITP included preventing severe bleeding episodes, maintaining a target platelet level >20–30 × 10^9^/L, minimizing treatment toxicity, and optimizing HRQoL (12). ITP management includes glucocorticoids, intravenous immune globulin, immunosuppressants, and occasionally platelet transfusions; withdrawal of anticoagulant and antiplatelet agents is advisable when the platelet count is low, <50 × 10^3^/L.

Thrombopoietin-receptor agonists (TPO-RAs) are used for patients who fail to respond to glucocorticoids or have recurrent bleeds and decrease in platelet count after glucocorticoids are discontinued (13). The TPO-RAs romiplostim, eltrombopag, and avatrombopag are approved by the Food and Drug Administration (FDA) and the European Medicines Agency (EMA) to treat patients with insufficient response to prior treatment. The three TPO-RAs are also approved by and commercially available in the Gulf Cooperation Council countries. These agents have demonstrated a high response rate in clinical trials including patients with chronic ITP (14–16). Moreover, long-term treatment with TPO-RAs was associated with a significant improvement in HRQoL, particularly romiplostim (17–19) and eltrombopag (20, 21).

The different molecular structures of TPO-RAs contribute to the differences in their pharmacodynamics and pharmacokinetics. For example, romiplostim is administered subcutaneously; however, avatrombopag and eltrombopag are administered orally rendering them vulnerable to food-drug interaction. Interaction with food (particularly polyvalent cations) is attributed to the biphenyl hydrazone structure in eltrombopag; hence, dietary restriction is required (22). Food-drug interaction is one of the challenges in oral administration as food may impact drugs' release, absorption, distribution, and metabolism. On the other hand, pharmacodynamic drug-food interaction results in a particular pharmacological effect (23).

Fasting was found to be one of the factors that affect drugs' metabolism and response (24). Knowing the effects of fasting contributes to predicting drug response and to optimizing disease management. This paper aims to review the current TPO-RAs approved for primary ITP (romiplostim, eltrombopag, avatrombopag) and the effect of intermittent fasting in adult patients with primary ITP receiving TPO-RAs. Hetrombopag (a novel TPO-RA approved only in China for the treatment of ITP) was also included in our review. Lusutrombopag (approved for thrombocytopenia in patients with chronic liver disease) is outside the scope of our review.

## 2. Methods

The authors conducted a literature search, with no limits on date or language, using various combinations of keywords, including “immune thrombocytopenia”, “thrombopoietin receptor agonist”, “intermittent fasting”, “avatrombopag”, “eltrombopag”, “romiplostim”, and “hetrombopag”. Further references were identified by searching the reference lists of retrieved articles and from the authors' knowledge of the field. The authors concluded the manuscript with their expert opinion.

## 3. Results

### 3.1. Romiplostim

#### 3.1.1. Pharmacokinetics and pharmacodynamics

The pharmacokinetics and pharmacodynamics of romiplostim were evaluated in a double-blind, single-dose study. Healthy subjects received a single dose of placebo, subcutaneous romiplostim (a dose range of 0.1–2 µg/kg), or intravenous romiplostim (a dose range of 0.3–10 µg/kg). The achieved biologically active dose (defined as more than two-fold increase in platelet count) was 2 µg/kg subcutaneously and 1 µg/kg intravenously (25). There was a dose-dependent increase in the platelet count, which increased 1–3 days after intravenous administration, 4–9 days after subcutaneous administration, and peaking on days 12–16 (25). The half-life ranged from 1 to 34 days, with a median of 3.5 days (26). Romiplostim is eliminated primarily by the platelets and the mononuclear phagocytic cells, and has a biphasic distribution that was nonlinear with dose (25). Since the elimination of romiplostim is platelet-dependent, clearance and serum concentration are partly and inversely dependent on patient's platelet count (27, 28). Such a wide half-life range may interfere with dose-response predictability and dosing schedule.

**Figure 1:**
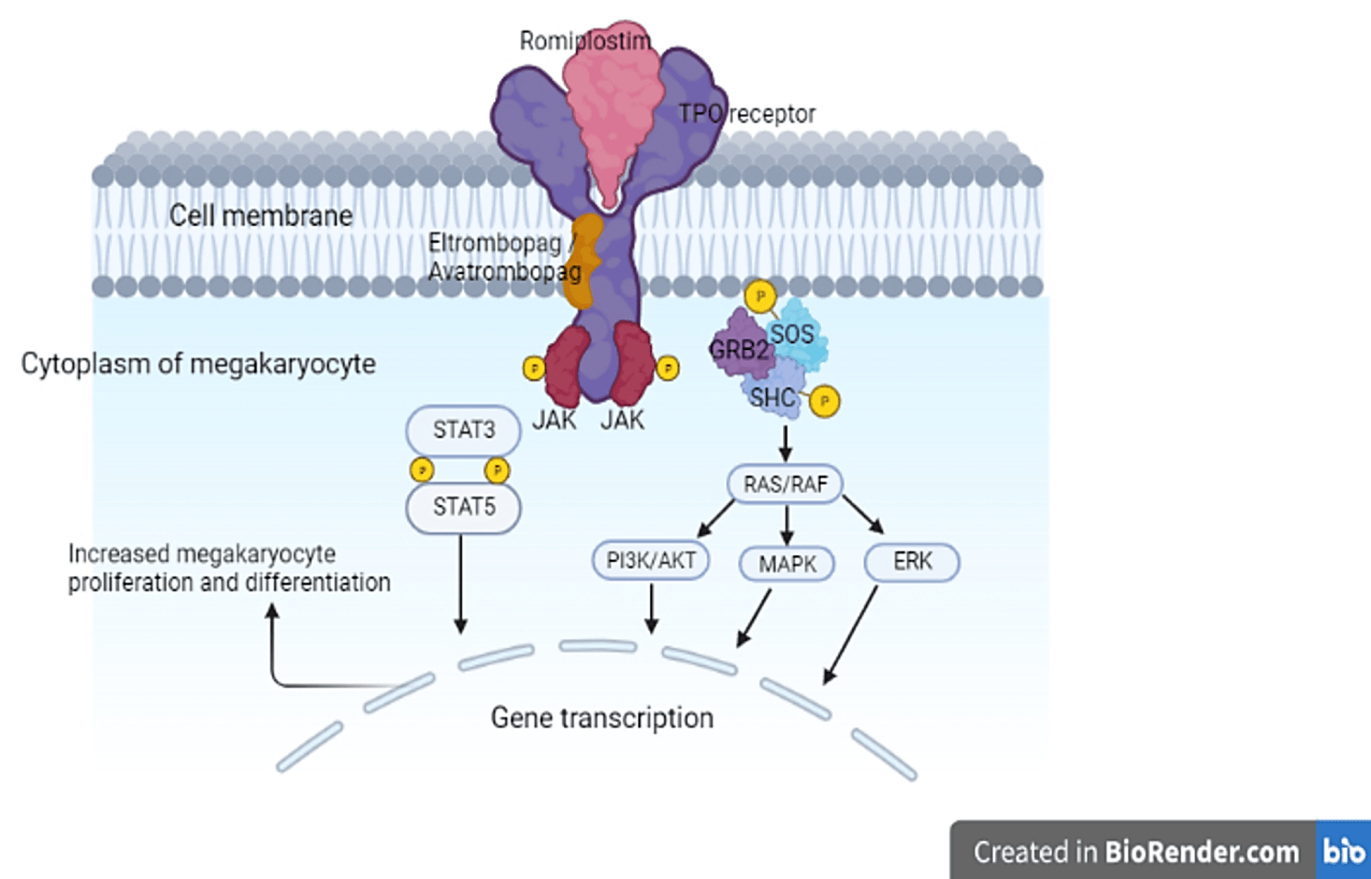
Mechanism of action of TPO-RAs. Binding of the TPO-RAs to the TPO receptor induces a conformational change in the receptor and the phosphorylation (activation) of the Janus kinases. Consequently, various signaling pathways are activated which result in increased platelet production. AKT, protein kinase B; ERK, extracellular-signal-regulated kinase; GRB2, growth factor receptor-binding protein 2; JAK, Janus kinase; MAPK, mitogen-activated protein kinase; P, phosphorylation; PI3K, phosphatidylinositol 3-kinase; RAF, rapidly accelerated fibrosarcoma kinase; RAS, rat sarcoma guanosine triphosphatase; SHC, Src homology collagen protein; SOS, son of sevenless; STAT, signal transducer and activator of transcription; TPO, thrombopoietin; TPO-RA, thrombopoietin-receptor agonist.

Romiplostim, a peptibody (a structure composed of a biologically active peptide fused to the Fc region of an antibody) (29), binds to and stimulates TPO receptors found on cells such as megakaryocytes, platelets, stem cells, and all progenitor cells found in the bone marrow. This binding activates intracellular transcriptional pathways, ultimately stimulating megakaryopoiesis (26) (Figure 1).

#### 3.1.2. Clinical efficacy and safety

A large body of evidence supports the efficacy and safety of romiplostim in treating ITP. Several clinical trials of different phases showed that romiplostim increased platelet count sustainably and reduced the rate of bleeding events in adults with ITP (16, 30–36). In addition, results from observational and real-world studies were similar to those of clinical trials; data showed that romiplostim improved platelet count and reduced bleeding events and hospitalization (37, 38). Interestingly, some studies reported that patients had maintained a treatment-free response for a considerable duration (33, 39–42).

In a phase 2 trial, median time from first dose to peak platelet count was 18 days (range, 8–43) for 1 μg/kg dose, 19 days (range, 8–36) for 3 μg/kg, and 63 days (range, 7–78) for placebo (30). In a phase 3 trial including both splenectomized and nonsplenectomized adult patients, a target platelet count of ≥50 × 10^9^/L was achieved by 25% of patients after 1 week and by 50% within 2–3 weeks of romiplostim administration (16). In another phase 3 trial, 76% of adults who received romiplostim responded (platelet count ≥50 × 10^9^/L) at Week 2 (31).

A pooled analysis reported that 68% of splenectomized patients and 80% of nonsplenectomized patients attained a sustained platelet response (defined as platelet counts ≥50 × 10^9^/L for 9 out of 12 weeks, with no use of rescue medication during the 4 weeks prior to each qualifying platelet count) (36).

In a prospective multicenter interventional study including adults with persistent or chronic primary ITP and complete response on TPO-RAs (romiplostim or eltrombopag), 27 of 48 patients achieved sustained response off treatment (platelet count >30 × 10^9^/L and no bleeding), and 15 of 48 patients achieved sustained complete response off treatment (platelet count >100 × 10^9^/L and no bleeding) at Week 24 (43). Moreover, in a multicenter observational study of 121 adult patients with ITP, 51.3% of patients receiving romiplostim achieved therapy-free response (platelet counts ≥50 × 10^9^/L for at least 6 months in the absence of any therapies meant to increase platelet counts) (42).

Analysis of data integrated from nine studies conducted between 2002 and 2014 demonstrated that romiplostim increased platelet counts in patients with either ITP ≤1 year or ITP >1 year, with more treatment-free remission in those with ITP ≤1 year. Both romiplostim and placebo/standard of care had comparable safety profiles (44).

ITP-patient assessment questionnaire was used to investigate the impact of romiplostim therapy on HRQoL in two, placebo-controlled, phase 3 clinical trials of splenectomized and non-splenectomized patients. Data pooled from these clinical trials showed that romiplostim significantly improved HRQoL in adult patients with chronic ITP (18).

Romiplostim was well-tolerated and showed mild to moderate adverse events (AEs) in clinical trials. Short-term AEs were headache, fatigue, arthralgia, dizziness, insomnia, myalgia, pain in the extremity, abdominal pain, shoulder pain, dyspepsia, and paresthesia (16, 31, 34, 35, 45). The reported serious AEs were bleeding, thrombosis, increased bone marrow reticulin, and hematologic cancer or myelodysplastic syndromes (31, 35, 46). Bleeding was not deemed to be treatment-related (35, 46). Although there was no increased risk of thromboembolic complications associated with romiplostim, it should be used cautiously in patients with ITP who have a history of, or are at increased risk of, thromboembolic complications (36, 45, 47–50).

Few studies reported that some patients developed neutralizing antibodies to romiplostim; however, these antibodies were not associated with a clinical complication or reduction in platelet count (51, 52).

### 3.2. Eltrombopag

#### 3.2.1. Pharmacokinetics and pharmacodynamics

The pharmacokinetics of eltrombopag were dose-dependent and linear (53). Its absorption is reduced when administered with products containing polyvalent cations (e.g., antacids), calcium-rich foods, and mineral supplements (iron, calcium, aluminum, magnesium, selenium, zinc), high-fat meals (54, 55). Therefore, it is recommended to administer eltrombopag under fasting conditions, with low-calcium foods only, or four hours after and two hours before food and products containing polyvalent cations (e.g., antacids, mineral supplements and dairy products) (56). Eltrombopag is mainly metabolized in the liver and no clinically significant interactions were detected when it is co-administered with cytochrome P450 (CYP) substrates, inducers, or inhibitors (55).

Eltrombopag is a small non-peptide molecule that binds to the transmembrane domain of the TPO receptor which is expressed on the surface of stem cells and megakaryocytes (57, 58). Binding to the TPO receptor activates intracellular signal transduction pathways that increase the proliferation and differentiation of human bone marrow progenitor cells leading ultimately to the production of normal platelets (59–61) (Figure 1). Eltrombopag interacts synergistically with endogenous TPO rather than competing for its binding site (62). In a phase 1 clinical trial, eltrombopag showed a consistent increase in platelet count starting after 8 days of repeated dosing with a peak platelet count achieved at day 16 (53).

#### 3.2.2. Clinical efficacy and safety

The efficacy and safety of eltrombopag were evaluated in several trials which are summarized in Table 1.

**Table 1:** Clinical trials evaluating eltrombopag in ITP patients.

Study	Design	Outcome
Bussel et al. (15)	Phase 2, multicenter, randomized, double-blind, placebo-controlled, dose-finding	Response rate was 28%, 70%, and 81% with 30, 50 and 75 mg/day doses respectively (placebo: 11%)
	Primary endpoint: platelet count greater than 50 × 10^3^/L on day 43	
Bussel et al. (63)	Phase 3, multicenter, randomized, double-blind, placebo-controlled	Response rate in eltrombopag group (59%) was significantly higher than that in placebo group (16%)
	Primary endpoint: platelet count greater than 50 × 10^9^/L on day 43	
Cheng et al. (RAISE) (20)	Phase 3, multicenter, randomized, double-blind, placebo-controlled	Number of responders at least once was significantly higher in the eltrombopag group (79%) than in the placebo group (28%)
	Primary endpoint: platelet count of 50–400 × 10^9^/L	
Bussel et al. (REPEAT) (64)	Phase 2, multicenter, open-label, single-arm, repeat-dose	Response rate in Cycle 1 was 80%, among whom 87% also responded in Cycle 2 or Cycle 3, and 71% maintained the response in both Cycles 2 and 3
	Primary endpoint: consistency of response (platelet count greater than 50 × 10^9^/L and two times the baseline) in all three cycles	
Saleh et al. (EXTEND) (21)	Multicenter, open-label extension study enrolling patients who completed the four studies reported above without having experienced eltrombopag-associated serious adverse events	Eltrombopag was well-tolerated and safe with an overall response of 88%
	Primary endpoints: safety and tolerability of eltrombopag	
	Secondary endpoint: platelet count of at least 50 × 10^9^/L at least once during treatment	

ITP, immune thrombocytopenia.

A dose-finding phase 2 trial was conducted to determine the optimal dose of eltrombopag. Patients with chronic ITP were randomized into four groups receiving 30, 50, and 75 mg of eltrombopag, or placebo daily for 6 weeks. The primary efficacy endpoint was a platelet count greater than 50 × 10^9^/L or more on day 43. The response rate was 70% and 81% in groups receiving 50 and 75 mg of eltrombopag respectively. It was significantly greater than that observed in the placebo group (11%) and sustained over the entire duration of treatment. The response rate in the 30 mg group (28%) did not differ significantly from that of the placebo group. Both the 50 and 75 mg groups showed a reduction in bleeding symptoms (15).

A phase 3 randomized, double-blind clinical trial was conducted where patients received either 50 mg of eltrombopag or a placebo for up to 6 weeks. The primary endpoint was similar to that used in the dose-finding study. The response rate in the eltrombopag group (59%) was significantly greater than that of the placebo group (16%). In addition, patients in the eltrombopag group showed fewer bleeding symptoms during the treatment duration. After treatment discontinuation, platelet counts returned to baseline in the following weeks and the percentage of patients presenting bleeding symptoms increased (63).

The RAISE trial was a double-blind phase 3 study evaluating the safety and efficacy of eltrombopag for a prolonged treatment duration (6 months) (20). The primary endpoint was the odds of reaching a platelet count of 50–400 × 10^9^/L. It was significantly greater in the eltrombopag group than in the placebo group throughout the 6-month treatment period. In addition, the rates of bleeding and clinically significant bleeding were significantly lower in the eltrombopag group. However, platelet counts returned to baseline after discontinuation of eltrombopag.

The REPEAT trial (an open-label, single-arm, repeat-dose phase 2 study) investigated the effectiveness of repeated short-term cycles of eltrombopag (64). Recruited patients received 50 mg of eltrombopag daily over 3 cycles, each consisting of up to 6 weeks on-therapy followed by up to 4 weeks off therapy. A response to treatment was defined as a platelet count of more than 50 × 10^9^/L and at least twice the baseline count at Day 43 of the treatment cycle, and the primary endpoint was the proportion of patients with a response in Cycle 1 who subsequently responded in Cycles 2 or 3. In Cycle 1, 80% of patients responded of whom 87% responded in Cycles 2 or 3% and 71% in Cycles 2 and 3.

The open-label EXTEND study evaluated long-term safety and efficacy of eltrombopag in adults with ITP who had completed a previous eltrombopag study (65). The primary endpoints were safety and tolerability parameters, including clinical laboratory tests, ocular examinations, and frequency of all AEs. The secondary endpoints included the proportion of patients who achieved platelet count of at least 50 × 10^9^/L at least once during treatment. The median duration of eltrombopag treatment was 2.4 years and the study follow up patients for around 8 years. Around 86% of patients achieved a platelet count of at least 50 × 10^9^/L at any time during the study period; more than half (52%) of patients achieving a count higher than 50 × 10^9^/L maintained a continuous platelet count of 50 × 10^9^/L or more for at least 25 weeks in the absence of rescue therapy.

A phase 2 trial explored the sustained remission off treatment achieved by adult patients with newly diagnosed or persistent primary ITP treated with eltrombopag. The primary endpoint was the proportion of subjects who achieved sustained remission off treatment defined as the proportion of responders that were able to taper and discontinue eltrombopag maintaining a platelet count of at least 30 × 10^9^/L during a period of observation of 24 weeks with no bleeding nor administration of other ITP medications. The rate of sustained remission off treatment was 25% in patients who started eltrombopag (66).

Overall, eltrombopag was well-tolerated and safe, and important AEs (thrombosis, hepatobiliary AEs, and bone marrow fibrosis) were infrequent. Headache was the most frequent AE in all eltrombopag trials. All studies showed no significant changes in blood coagulation and platelet aggregation, and electrocardiographic findings (QT prolongation) (55, 67). Eltrombopag use is associated with elevations in alanine aminotransferase and bilirubin which may resolve despite ongoing treatment (20). The safety profile of eltrombopag is satisfactory. However, patients should be closely monitored for thromboembolic events or hepatic damage (68).

### 3.3. Avatrombopag

#### 3.3.1. Pharmacokinetics and pharmacodynamics

Avatrombopag is administered orally. After a single-dose administration, the increase in platelet count was dose-dependent, the maximum concentration was achieved within 6–8 h, and the half-life was 16–19 h (69). Avatrombopag is metabolized mainly via the hepatic enzymes CYP2C9 and CYP3A4 (70). It is mainly excreted through feces (88%) while only 6% is excreted in urine (69). Unlike eltrombopag (dietary restriction is required around the dose), dietary fat and divalent cations (e.g., calcium) do not impact the absorption of avatrombopag (22, 69). The administration of avatrombopag with meals reduces pharmacokinetic variability and does not affect the rate or extent of its absorption (69).

*In vitro* and *in vivo* studies showed that avatrombopag mimics, to a certain extent, the effect of endogenous TPO through stimulating megakaryocyte development and megakaryocyte colony-formation from human CD34 + hematopoietic cells. Further studies of avatrombopag in humans have shown that a single dose of the drug leads to a dose-dependent increase in platelet count, which reached its maximum from baseline in 8–11 days, then returned to baseline at approximately 4 weeks after the dose (69). Avatrombopag works synergistically with endogenous TPO. For example, combining avatrombopag in with recombinant human TPO increased platelet count by up to 200% more than TPO alone (71).

#### 3.3.2. Clinical efficacy and safety

A phase 2 double-blind, randomized dose-ranging, placebo-controlled parallel-group study recruited patients to randomly receive either a once-daily fixed-dose of avatrombopag (2.5 mg, 5 mg, 10 mg, or 20 mg) or placebo and received treatment for 28 days (72). The primary endpoint was a platelet count response defined as the proportion of patients who achieved platelet count ≥50 × 10^9^/L and a minimum increase of 20 × 10^9^/L above baseline at day 28. The response rates of the avatrombopag group were 13%, 53%, 50%, and 80% in the 2.5, 5, 10, and 20 mg groups, respectively, compared to a 0% response rate in the placebo group. The objective of the extension part of the study was to assess the safety and tolerability of avatrombopag. Results of the extension part showed that 52% and 76% had a durable (platelet count response at ≥75% of their platelet assessments over the last 14 weeks) and overall (stable response or response at any ≥2 consecutive visits) response, respectively.

A second study was conducted to evaluate the efficacy and safety of avatrombopag in chronic ITP patients (14). It was a 6-month multicenter phase 3 trial; adult patients were randomly assigned to receive either 20 mg of avatrombopag daily or a placebo. The primary endpoint was the cumulative number of weeks with a platelet count of at least 50 × 10^9^/L without rescue therapy. It was significantly longer in the avatrombopag group (mean of 12 weeks) compared to the placebo group (mean of 0.1 weeks). Efficacy was sustained in the long-term extension part of the study. A *post hoc* analysis was conducted to evaluate the durability of platelet count response to avatrombopag in different subgroups of patients who were enrolled in this phase 3 multicenter trial. The analysis showed that response to avatrombopag was stable and durable despite having some variation in patients' characteristics (73).

In the two studies, avatrombopag appeared to be safe and tolerable. The most frequent AEs reported in the phase 2 trial were epistaxis, headache, fatigue, and confusion (72). On the other hand, the most common treatment-emergent AE reported in the phase 3 trial were headache, contusion, upper respiratory tract infection, arthralgia, epistaxis, fatigue, gingival bleeding, and petechiae (14).

The clinical characteristics and outcomes following avatrombopag initiation in adult ITP patients were described in a retrospective study conducted in the USA. In this recent real-world study, ITP patients achieved clinically meaningful platelet count with avatrombopag without the need of rescue medication; many were able to discontinue baseline concomitant steroid or immunosuppressants (74).

### 3.4. Hetrombopag

#### 3.4.1. Pharmacokinetics and pharmacodynamics

Oral hetrombopag demonstrated a high inter-individual pharmacokinetic variability in both healthy subjects and ITP patients. In healthy subjects, hetrombopag plasma concentration showed two peaks, and bioavailability was markedly reduced by high-fat and high-calorie food. Consequently, hetrombopag should be administered on an empty stomach to avoid drug-food interaction which ultimately decrease pharmacodynamic activity. The administration of hetrombopag in ITP patients showed a prolonged absorption and steady state was achieved around Day 10. The elimination half-life ranged between 23.2 and 39.8 h. Hetrombopag is extensively metabolized and the predominant route of excretion is via feces (89.05%) (75–78).

Hetrombopag is an oral non-peptide TPO-RA. Similar to eltrombopag and avatrombopag, hetrombopag binds to the transmembrane domain of human TPO receptor on progenitor cells and induces megakaryopoiesis. Hence, hetrombopag does not compete with the action of native thrombopoietin. The interaction between hetrombopag and the transmembrane domain of human TPO receptor activates intracellular thrombopoietin signaling pathways which ultimately induce platelet proliferation (78, 79).

#### 3.4.2. Clinical efficacy and safety

The efficacy and safety of hetrombopag in ITP patients were evaluated in a randomized, multicenter, placebo-controlled phase 3 study. Patients were randomized to receive 2.5 mg or 5 mg (to a maximum dose of 7.5 mg) hetrombopag once daily, or placebo for 10 weeks. The primary endpoint was the proportion of responders defined as patients achieving a platelet count of ≥50 × 10^9^/L after 8 weeks of treatment. The primary endpoint was significantly achieved in the 2.5 mg hetrombopag group (58.9%) and the 5 mg hetrombopag group (64.3%) vs. the placebo group (5.9%) (*p* < 0.0001). Platelet response to hetrombopag was durable and maintained after the 14-week extension period (80).

After the 10-week treatment period, the most common adverse events were upper respiratory tract infection, urinary tract infection, elevated platelet counts, hematuria, increased blood level of lactate dehydrogenase, and diarrhea. The reported serious adverse events were thrombocytopenia, gastrointestinal hemorrhage, and cerebral hemorrhage. However, none of the hemorrhagic episodes in the hetrombopag group were treatment-related (80).

Hetrombopag is currently approved in China for the treatment of primary ITP in adult patients who have failed to respond to other lines of treatment such as glucocorticoids and immunoglobulins (81).

### 3.5. Effect of intermittent fasting on TPO-RAs

Intermittent fasting is the voluntary abstinence from food and drinks for an extended period (e.g., 16–48 h) with intervening periods of regular food intake, on a recurring basis (82, 83). Different types of intermittent fasting were shown to impact health outcomes such as complete alternate-day fasting, time-restricted feeding, and religious fasting (e.g., Ramadan Fasting) (82).

Fasting affects the four processes of pharmacokinetics: absorption, distribution, metabolism, and excretion (84). A fast-break meal rich in carbohydrates, fats, and proteins may impact the rate and extent of drug absorption and elimination (84). For instance, the ability of eltrombopag to chelate with polyvalent cations, reduced plasma eltrombopag exposure by 70% when co-administered with an antacid containing metal cations (aluminum hydroxide and magnesium carbonate) (22).

The administration of romiplostim is considered convenient for ITP patients since it is given as a once-weekly subcutaneous injection with no dietary or time restriction (85). In addition, romiplostim has no drug-food interaction (3) and can be considered for ITP patients during Ramadan fasting, particularly if home self-injections are available (86). On the other hand, romiplostim may be inconvenient for some patients since, upon treatment initiation, more frequent clinic visits are required until platelet counts are stabilized. In addition, some patients may prefer oral route over injections (86).

Eltrombopag is an oral TPO-RA. However, it showed significant drug-food interaction; its bioavailability was reduced by 70% with aluminum hydroxide and magnesium carbonate antacid tablets compared to the fasting state (22). The bioavailability of eltrombopag was also reduced by 60% when administered with high-calcium dairy products, irrespective of fat content. The reduced bioavailability of eltrombopag will diminish its effect on platelet count. Therefore, patients should be aware of products containing metal cations (such as calcium, aluminum, iron, magnesium, selenium, or zinc) which should be taken at a different time of the day spaced from eltrombopag (22).

Eltrombopag must be administered on an empty stomach; it should be taken four hours after and two hours before food or products containing polyvalent cations (56). Some studies recommended a 4- to 6-hour fasting window around the administration of eltrombopag to separate it from polyvalent cations (e.g., antacids, dairy products, multivitamins) (22, 87). A retrospective study evaluating the effect of Ramadan fasting on patients with ITP receiving eltrombopag showed that platelet count dropped significantly during fasting and returned back to normal after Ramadan ended (88). In daily practice, patients may consider administering eltrombopag before bedtime during Ramadan fasting. Hence, healthcare providers must be aware of such practice as it may affect platelet count and increase bleeding risk (88).

Avatrombopag is another orally administered TPO-RA. Unlike eltrombopag, avatrombopag has no drug-food interaction and does not require dietary restriction around its administration (12, 89). Different studies showed that the administration of avatrombopag with food did not affect the rate or extent of its absorption; however, administration with food reduced intra- and inter-subject variability (69, 70). Therefore, it is recommended to administer avatrombopag with food (90). Interestingly, a study showed that around 51% of patients had switched from eltrombopag to avatrombopag due to its convenient administration without any dietary restrictions (89).

## 4. Conclusion/expert opinion

In general, TPO-RAs (either romiplostim or eltrombopag) are considered a second-line therapy for steroid-unresponsive chronic ITP patients (91). In the absence of head-to-head comparisons, there is no rationale to rank among these TPO-RAs. However, for fasting ITP patients in particular, we suggest that eltrombopag is not the preferred choice because drug-food interaction affects its bioavailability. In addition, the consequent dietary restriction creates a challenge for eltrombopag patients regarding adherence and quality of life. For instance, after being fasted for several hours (e.g., Ramadan fasting), it may not be convenient to administer eltrombopag with the fast-break meal.

Switching between TPO-RAs is a useful strategy to tackle some associated limitations including route of administration and dietary restriction. Romiplostim and avatrombopag have an advantage over eltrombopag as they do not require any dietary restrictions. We advise our patients with intermittent fasting to switch to romiplostim or avatrombopag if available. Since avatrombopag is administered orally, it may be preferred over romiplostim, injected subcutaneously. On the other hand, unlike romiplostim, avatrombopag is administered daily and not weekly. The choice of either drug will be based eventually on the patient's convenience and adherence.

In addition to fasting, many patients have erratic lifestyles because of shift working patterns, international travel and changes in time zones, and teenagers eating late at night; for these individuals, a treatment where strict timing of administration is required will often fail and compliance issues arise. These groups would also benefit from a convenient medication with no dietary restriction or refrigeration such as avatrombopag.

**Figure 2:**
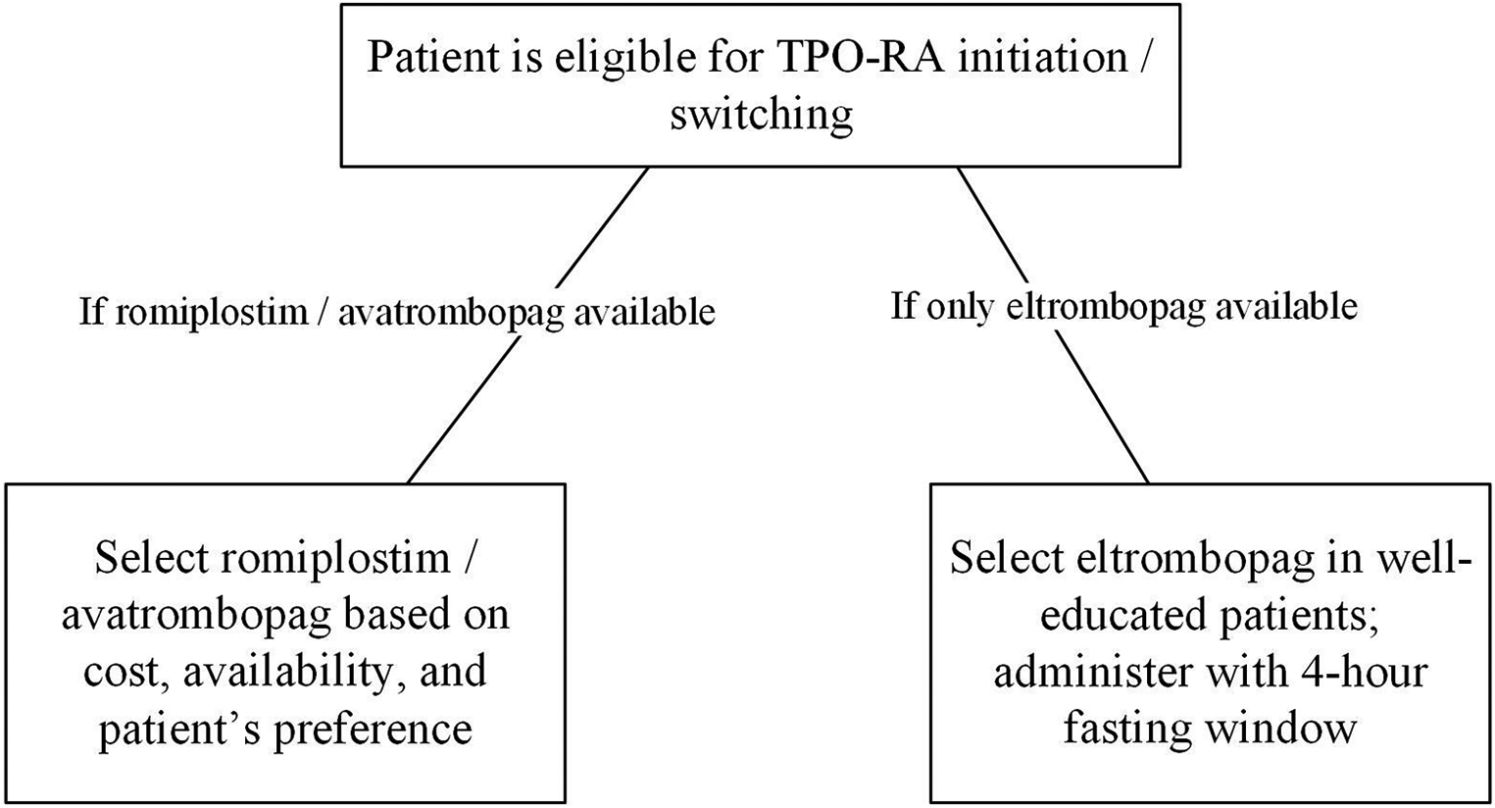
TPO-RAs selection in ITP patients. ITP, immune thrombocytopenia; TPO-RA, thrombopoietin-receptor agonist.

Since the three TPO-RAs have comparable safety and efficacy profiles, drug cost and availability could interfere in drug selection. In cases where romiplostim and avatrombopag are unavailable, patients should be educated on the appropriate administration, possible interactions, and dietary restrictions before initiating eltrombopag. Eltrombopag should be administered with a 4-hour fasting window to achieve optimal disease outcomes (Figure 2).
